# Prolonged Glucocorticoid Treatment in ARDS: Impact on Intensive Care Unit-Acquired Weakness

**DOI:** 10.3389/fped.2016.00069

**Published:** 2016-08-02

**Authors:** Gianfranco Umberto Meduri, Andreas Schwingshackl, Greet Hermans

**Affiliations:** ^1^Division of Pulmonary, Critical Care, and Sleep Medicine, Department of Medicine, Memphis Veterans Affairs Medical Center, Memphis, TN, USA; ^2^Department of Pediatrics, Division of Critical Care Medicine, Mattel Children’s Hospital at UCLA, Los Angeles, CA, USA; ^3^Laboratory of Intensive Care Medicine, Division of Cellular and Molecular Medicine, KU Leuven and Medical Intensive-Care Unit, Department of General Internal Medicine University Hospitals Leuven, Leuven, Belgium

**Keywords:** acute respiratory distress syndrome, glucocorticoid treatment, intensive care unit-acquired weakness, mechanical ventilation, survival, steroids, muscle weakness

## Abstract

Systemic inflammation and duration of immobilization are strong independent risk factors for the development of intensive care unit-acquired weakness (ICUAW). Activation of the pro-inflammatory transcription factor nuclear factor-κB (NF-κB) results in muscle wasting during disuse-induced skeletal muscle atrophy (ICU bed rest) and septic shock. In addition, NF-κB-mediated signaling plays a significant role in mechanical ventilation-induced diaphragmatic atrophy and contractile dysfunction. Older trials investigating high dose glucocorticoid treatment reported a lack of a sustained anti-inflammatory effects and an association with ICUAW. However, prolonged low-to-moderate dose glucocorticoid treatment of sepsis and ARDS is associated with a reduction in NF-κB DNA-binding, decreased transcription of inflammatory cytokines, enhanced resolution of systemic and pulmonary inflammation, leading to fewer days of mechanical ventilation, and lower mortality. Importantly, meta-analyses of a large number of randomized controlled trials investigating low-to-moderate glucocorticoid treatment in severe sepsis and ARDS found no increase in ICUAW. Furthermore, while the ARDS network trial investigating methylprednisolone treatment in persistent ARDS is frequently cited to support an association with ICUAW, a reanalysis of the data showed a similar incidence with the control group. Our review concludes that in patients with sepsis and ARDS, any potential direct harmful neuromuscular effect of glucocorticoids appears outweighed by the overall clinical improvement and reduced duration of organ failure, in particular ventilator dependency and associated immobilization, which are key risk factors for ICUAW.

Intensive care unit (ICU)-acquired weakness (ICUAW) is a condition that can either affect the peripheral nerves [critical illness polyneuropathy (CIP)], the skeletal muscle [critical illness myopathy (CIM)], or both and involves functional or structural changes in these tissues (1). Systemic inflammation (increased inflammatory cytokine levels) and associated organ dysfunction during early critical illness are strong independent risk factors for the development of CIM (2). As inflammatory infiltrates are rarely seen in muscle and nerve tissue in patients with ICUAW, the functional and structural damage may not be caused directly by inflammatory cell infiltration and activation, but rather stem from cytokine-driven electrophysiological alterations, endothelial and microvascular alterations, metabolic changes, and bio-energetic failure, ultimately leading to axonal cell death, pronounced muscle atrophy, and contractile dysfunction (3, 4).

In ARDS, inadequate GC-glucocorticoid receptor α (GRα)-mediated downregulation of the pro-inflammatory transcription factor nuclear factor-κB (NF-κB) in circulating and tissue-resident cells leads over time (>4 weeks) to a persistent elevation in plasma inflammatory cytokine levels indicating dysregulated systemic inflammation (5). NF-κB signaling is also involved in muscle wasting during disuse-induced skeletal muscle atrophy (ICU bed rest) and septic shock (6) and is a critical regulator of the catabolic response to TNFα (7). NF-κB activation also plays a significant signaling role in mechanical ventilation-induced diaphragmatic atrophy and contractile dysfunction by increasing transcription of specific atrophy-related genes (8). Hence, dysregulated systemic inflammation not only leads to delayed resolution of ARDS (5) but also contributes to the development of ICUAW.

Another important risk factor for ICUAW is the duration of immobilization (9, 10). ICUAW is often associated with respiratory muscle weakness (11). The phrenic nerve and diaphragm show similar electrophysiological and anatomo-pathological abnormalities as the peripheral nerves and muscles, contributing to delayed weaning from mechanical ventilation (12, 13). In turn, prolonged duration of mechanical ventilation itself can further exacerbate the weakness and atrophy of the diaphragm, which is often the clinical problem with which these patients present (10, 14). In addition to prolonged mechanical ventilation, ICUAW is also associated with longer ICU stay, increased mortality (3) as well as with long-term consequences beyond the hospitalization phase, which contribute substantially to the long-term financial burden of ARDS survivors on the health-care budget (10, 15, 16).

Although myopathy is a well-known adverse effect of chronic glucocorticoid administration (17), it is important to recognize that in fact glucocorticoids could potentially downregulate detrimental inflammatory pathways involved in ICUAW (1) and provide a protective effect if hyperglycemia is controlled (18). Experimental ARDS is associated with a significant reduction in lung tissue GRα expression (19–21) and increase in GRβ mRNA (20) leading to decreased GRα nuclear translocation (20). In these experiments, low-dose glucocorticoid treatment, contrary to placebo, restored GRα number and function leading to the resolution of pulmonary inflammation (21, 22). In an *ex vivo* ARDS study, prolonged methylprednisolone treatment, contrary to placebo, was associated with upregulation in GRα, significant increases in GC–GRα-mediated activities (GRα binding to NF-κB, GRα binding to GC response element on DNA, stimulation of inhibitory protein IκBα, and stimulation of IL-10 transcription), and significant reductions in NF-κB–κb DNA-binding and the transcription of TNF-α and IL-1β (23). ARDS patients randomized to prolonged methylprednisolone treatment, contrary to placebo, demonstrated a rapid and sustained reduction in markers of systemic inflammation (23, 24).

Recent guidelines (25, 26) and reviews (27) have provided an incomplete representation of the available evidence on glucocorticoid treatment in ARDS by citing imprecise meta-analysis (28) or contradictory results among meta-analyses (28, 29). This is having significant repercussions for the care of patients with ARDS and necessitates a clarification. A meaningful analysis of glucocorticoid treatment in ARDS must be founded on present understanding of disease pathophysiology and fundamentals of pharmacological treatment (5). The beneficial effects of glucocorticoid therapy in sepsis and ARDS are affected by four critical components of therapy: timing of initiation, dosage, duration of treatment, and tapering. In the 1980s, based on a faulty laboratory model (30), clinical investigations focused on a 1-day course of massive doses of methylprednisolone for prevention (31) or treatment of ARDS (32). Patients received 120 mg/kg of methylprednisolone (31, 32), equivalent to ~1-year of prednisone 20 mg daily. The prevailing fundamental principle (1950–1980) that treatment should be continued until disease resolution was omitted from the design of these negative trials (30). The findings of these randomized controlled trials (RCTs) halted the progress of the field (30). Surprisingly, these obsolete trials are often combined with contemporary trials in meta-analyses, despite serious inconsistency (28, 33) often producing misleading results. Furthermore, these meta-analyses also include selected retrospective cohort studies increasing the risk for imprecision (34, 35). Over the last 20 years, RCTs have instead investigated only low-to-moderate daily dosage (≤1 mg/kg for early ARDS and ≤2 mg/kg of methylprednisolone-equivalent for late ARDS) for 1–4 weeks; meaningful meta-analyses should focus on these RCTs that are relevant today.

Our recent systematic review (36) included trial and patient-level meta-analyses of eight RCTs (*n* = 619) investigating prolonged low-dose glucocorticoid treatment in ARDS. With high certainty, glucocorticoids improved time to extubation (10.1 fewer days, 95% confidence interval −13.1 to −7.1) and mechanical ventilation-free days at day 28 (5.8 more days, 95% confidence interval 3.8–11.5) and with moderate certainty, reduced in-hospital mortality by 24% (95% confidence interval 2–41%), for those randomized before day 14 of ARDS. Importantly, avoiding sudden discontinuation of methylprednisolone administration after extubation is essential to preserve improvement (36). For patients randomized after day 13 of ARDS, the ARDS network original report (37) found increased 60-day mortality. However, this subgroup (*n* = 48) had an uncharacteristically low mortality (8%) and large differences in baseline characteristics (38). When the analysis was adjusted for the imbalances at baseline, the mortality difference lost significance (25.6 vs. 13.2%; *p* = 0.325) (39). The results show that our meta-analysis (36) is also consistent with a recent meta-analysis of 13 RCTs (*n* = 2,005) investigating low-dose glucocorticoid treatment in community-acquired pneumonia (leading cause of ARDS) (40). In addition to a potential reduction in mortality, glucocorticoid treatment reduced the risk of progression to ARDS (relative risk 0.24, 95% confidence interval 0.10–0.56) or need for mechanical ventilation (relative risk 0.45, 95% confidence interval 0.26–0.79) – both moderate certainty (40).

Although some reports identified glucocorticoid administration as a significant risk factor for ICUAW (12, 41) or short- to medium-term functional outcome (42, 43), most were unable to demonstrate an association between prolonged low-to-moderate dose glucocorticoid treatment and electrophysiologically or clinically proven neuromuscular dysfunction (2, 15, 18, 44–48) or with any physical outcome at discharge and up to 2 years following ARDS (15). Meta-analyses limited to randomized trials investigating prolonged glucocorticoid treatment in ARDS (29) and sepsis (49) have reported a similar rate of ICUAW in the control and treated groups. Nevertheless, the ARDS network trial (37) is frequently cited for concluding that methylprednisolone is associated with a higher rate of neuromuscular weakness. However, the actual results of the trial (37) show a similar rate (placebo vs. methylprednisolone) of weakness [21 (22%) vs. 26 (30%); *p* = 0.20] and similar 60-day mortality (ARDSnet02 Dataset) for those with weakness [3 (14%) vs. 2 (8%)]. In a subsequent publication (45), after removal of six patients (five randomized to methylprednisolone) with documented weakness at study entry, the ARDS network authors confirmed that methylprednisolone was not associated with increased incidence of weakness [21 (24%) vs. 20 (22%)]. Importantly, in the Appendix of the original publication (37), Table 7 showed that median duration (days) of MV (placebo vs. methylprednisolone) was appreciably lower for those randomized to methylprednisolone with [26 (14–38) vs. 15 (12–22)] or without weakness [16 (9–30.5) vs. 8.5 (5–21)], respectively.

Furthermore, the original ARDS network publication (37) placed much attention to a subgroup of methylprednisolone patients with weakness reported as a serious adverse event (0 vs. 9; *p* < 0.001). An adverse event or suspected adverse reaction must be reported to the sponsor within 72 h of identification and is considered “serious” (SAE) if, in the view of either the investigator or sponsor, it results or may result in a serious outcome, such as death or prolongation of existing hospitalization (50). However, the methylprednisolone patients in question did not have increased duration of mechanical ventilation or death in comparison to the control group. The methylprednisolone-treated patients with SAE related to weakness had a median duration on initial mechanical ventilation of 16.5 days (7–28) and only one death. The death occurred in a patient with high severity indices (APACHE III 99, SOFA score 14) with study drug removed without tapering the day after successful extubation (study day 14) that returned to MV 3 days later (without reinstitution of study drug in the presence of possible adrenal suppression) and died on study day 20 (ARDSnet02 Dataset).

In summary, while ICUAW is an independent predictor of prolonged weaning (51) and is associated with increased mortality, randomized ARDS trials investigating prolonged low-dose glucocorticoid treatment have consistently reported a sizable and significant increase in mechanical ventilation-free days and reduction in hospital mortality for those randomized before day 14 (36). Based on these observations, it is reasonable to conclude that glucocorticoid treatment-associated downregulation of systemic inflammation in ARDS does not induce clinically relevant neuromuscular dysfunction. Alternatively, any potential direct harmful neuromuscular effect of glucocorticoids appears outweighed by the overall clinical improvement and reduced duration of organ failure, in particular ventilator dependency and associated immobilization, which are key risk factors for ICUAW.

## Authors Contributions

The authors co-wrote, reviewed, and approved the final report.

## Conflict of Interest Statement

The authors have no competing interests to declare or any real or perceived financial interest in any product or commodity mentioned in this paper.
